# Normal imaging findings after aortic valve implantation on ^18^F-Fluorodeoxyglucose positron emission tomography with computed tomography

**DOI:** 10.1007/s12350-019-02025-y

**Published:** 2020-01-23

**Authors:** Ali R. Wahadat, Wilco Tanis, Asbjørn M. Scholtens, Margreet Bekker, Laura H. Graven, Laurens E. Swart, Annemarie M. den Harder, Marnix G. E. H. Lam, Linda M. de Heer, Jolien W. Roos-Hesselink, Ricardo P. J. Budde

**Affiliations:** 1 Department of Radiology and Nuclear Medicine, Erasmus Medical Center, Rotterdam, The Netherlands; 2 Department of Cardiology, Thoraxcenter, Erasmus Medical Center, Rotterdam, The Netherlands; 3 Department of Cardiology, Haga Teaching Hospital, The Hague, The Netherlands; 4 Department of Nuclear Medicine, Meander Medical Center, Amersfoort, The Netherlands; 5 Department of Thoracic Surgery, Thoraxcenter, Erasmus Medical Center, Rotterdam, The Netherlands; 6 Department of Radiology and Nuclear Medicine, Utrecht Medical Center, Utrecht, The Netherlands; 7 Department of Cardiothoracic Surgery, Utrecht Medical Center, Utrecht, The Netherlands; 8 Department of Cardiothoracic Surgery, Leiden University Medical Center, Leiden, The Netherlands; 9 Department of Radiology and Nuclear Medicine, Erasmus MC, ND-547, Dr. Molewaterplein 40, 3015GD Rotterdam, The Netherlands

**Keywords:** Infection, Valvular heart disease, PET, Inflammation, Image interpretation

## Abstract

**Background:**

To determine the normal perivalvular ^18^F-Fluorodeoxyglucose (^18^F-FDG) uptake on positron emission tomography (PET) with computed tomography (CT) within one year after aortic prosthetic heart valve (PHV) implantation.

**Methods:**

Patients with uncomplicated aortic PHV implantation were prospectively included and underwent ^18^F-FDG PET/CT at either 5 (± 1) weeks (group 1), 12 (± 2) weeks (group 2) or 52 (± 8) weeks (group 3) after implantation. ^18^F-FDG uptake around the PHV was scored qualitatively (none/low/intermediate/high) and quantitatively by measuring the maximum Standardized Uptake Value (SUV_max_) and target to background ratio (SUV_ratio_).

**Results:**

In total, 37 patients (group 1: n = 12, group 2: n = 12, group 3: n = 13) (mean age 66 ± 8 years) were prospectively included. Perivalvular ^18^F-FDG uptake was low (8/12 (67%)) and intermediate (4/12 (33%)) in group 1, low (7/12 (58%)) and intermediate (5/12 (42%)) in group 2, and low (8/13 (62%)) and intermediate (5/13 (38%)) in group 3 (*P* = 0.91). SUV_max_ was 4.1 ± 0.7, 4.6 ± 0.9 and 3.8 ± 0.7 (mean ± SD, *P* = 0.08), and SUV_ratio_ was 2.0 [1.9 to 2.2], 2.0 [1.8 to 2.6], and 1.9 [1.7 to 2.0] (median [IQR], *P* = 0.81) for groups 1, 2, and 3, respectively.

**Conclusion:**

Non-infected aortic PHV have similar low to intermediate perivalvular ^18^F-FDG uptake with similar SUV_max_ and SUV_ratio_ at 5, 12, and 52 weeks after implantation.

**Electronic supplementary material:**

The online version of this article (10.1007/s12350-019-02025-y) contains supplementary material, which is available to authorized users.

## Introduction

Diagnosing prosthetic heart valve (PHV) endocarditis remains difficult.^1^,^2^^18^F-Fluorodeoxyglucose (^18^F-FDG) Positron Emission Tomography (PET) with computed tomography (CT) was added as an additional diagnostic tool in the 2015 European Society of Cardiology (ESC) guidelines for infectious endocarditis.^2^ Since then, ^18^F-FDG PET/CT has shown great potential for diagnosing PHV endocarditis, with a good sensitivity and specificity.^3^–^5^ For accurate interpretation of ^18^F-FDG PET/CT scans in PHV patients suspected for endocarditis, knowing the normal amount and pattern of ^18^F-FDG uptake around PHV’s (due to the normal tissue healing response) is important. The ESC guidelines suggest using ^18^F-FDG PET/CT only if the PHV was implanted > 3 months prior to the scan because it was assumed that the normal healing response after aortic PHV implantation and its associated ^18^F-FDG uptake would cause false positive results and misinterpretations within this time window.^2^ However, this arbitrary time period is not based on any evidence and has recently been questioned in other studies.^3^,^6^ Indications of the normal ^18^F-FDG uptake patterns and cut-off values for abnormal uptake have been obtained from retrospective assessment of a limited number of patients with a PHV who underwent ^18^F-FDG PET/CT for indications other than suspected endocarditis.^3^,^7^ Recently, the first prospective study regarding baseline assessment of normal ^18^F-FDG uptake patterns around PHV’s was published showing no significant differences between ^18^F-FDG uptake around PHV’s at 1, 6 and 12 months after surgery.^8^ In this study, we prospectively assessed the qualitative and quantitative baseline perivalvular ^18^F-FDG uptake at three different time points within the first year following aortic PHV implantation, in order to obtain normal ^18^F-FDG uptake reference values.

## Materials and Methods

### Patient Selection and Classification

In this prospective multi-center cross-sectional study, we included patients (age ≥ 50 years) from two different hospitals in the Netherlands (Erasmus Medical Center, Rotterdam, and the University Medical Center, Utrecht) who had undergone an uncomplicated aortic PHV implantation. An uncomplicated PHV implantation was defined as a PHV implantation without any surgical complication during or after the operation as well as the absence of signs of infection as mentioned in the surgical reports and the electronic patient files. The inclusion and exclusion criteria are detailed in Table 1. The medical ethics committee approved the study (NL42743.041.12). All patients provided written informed consent.

**Table 1:** Inclusion and exclusion criteria

Inclusion criteria	Exclusion criteria
Age ≥ 50 years Patients after uncomplicated PHV implantation in aortic position (mechanical and biological PHVs) Normal routine follow up TTE (standardly performed 5 days after operation) or intra-operative TEE. With no signs of obstruction, endocarditis or significant paravalvular leakages Weight < 110 kg	Known contrast allergy Known renal impairment (according to local hospital guidelines) Diabetes Mellitus Mild contractile dysfunction of the left and/or right ventricle (Eyeballing, Ejection fraction < 45 %, TAPSE < 14 mm) Active cardiac decompensation Uncontrolled cardiac arrhythmias Suspicion of active endocarditis Previous participation in scientific studies using radiation (Possible) pregnancy in pre-menopausal women above 50 years not on reliable birth control therapy. Other contraindications for contrast use according to the standard daily clinical routine according to the protocol by the department of radiology Use of pericardial patches and re-operation of aortic PHV in past medical history Contraindication for Computed Tomography Angiography according the standard daily clinical routine Refusal to be informed about potential additional CT or FDG PET findings If already included in group 1, patients cannot be included in group 2 or 3

*PHV*, prosthetic heart valve; *TTE*, transthoracic echocardiogram; *TEE*, transesophageal echocardiogram; *TAPSE*, tricuspid annular plane systolic excursion; *CT*, computed tomography; *FDG PET*, fluorodeoxyglucose positron emission tomography

Patients were divided into three groups and received an ^18^F-FDG PET/CT at either 5 (± 1) weeks (group 1), 12 (± 2) weeks (group 2), or 52 (± 8) weeks (group 3) following valve implantation. The assignment of patients to each group depended on logistic factors such as availability of time slots on the PET/CT scanner and patient availability of one of the three time intervals after surgery.

Included patients had undergone uncomplicated valve implantations and did not have any clinical signs of prosthetic valve infection (fever, shivers, dyspnea, etc) at the time of the ^18^F-FDG PET/CT.

### Image Acquisition

#### ^18^F-FDG PET/CT

To induce free fatty acid metabolism and suppress myocardial glucose metabolism, patients followed a 24-hour low carbohydrate diet, of which the last 12 hours were spent fasting.^9^–^11^ Thereafter, patients received an intravenous ^18^F-FDG injection of 2.0 MBq/kg. Patients were hydrated with 1000 ml of water 1 hour prior to image acquisition. Blood glucose levels were checked before ^18^F-FDG injection and the limit was set to 8.9 mmol/L. Approximately 1 hour after ^18^F-FDG injection, the PET/CT was performed using a Biography Sensation 16scanner (SIEMENS Medical, Germany). Before the PET acquisition, a low dose CT scan was performed for attenuation correction. A PET-scan of the heart was then obtained with 3-minute acquisitions per bed position using a 3-dimensional acquisition mode. Attenuation-corrected PET images were reconstructed with an ordered-subset expectation-maximization iterative reconstruction algorithm.

### Image Analysis and Interpretation

#### ^18^F-FDG PET/CT analysis

Uptake of ^18^F-FDG around the PHV was scored both qualitatively and quantitatively by an experienced nuclear medicine physician. For qualitative analyses, the Qualification Visual Score for Hypermetabolism (QVSH) was used, scoring the uptake as “none” (no or less than mediastinal uptake), “low” (more than mediastinal uptake but less than in the liver), “intermediate” (more than liver uptake), or “high” (intense uptake). Mediastinal uptake was defined as the mean uptake in the blood pool of the descending aorta at the level of the left atrium. Additionally, the location (former left coronary cusp (LCC)/right coronary cusp (RCC)/non coronary cusp (NCC), circular, PHV struts only or ascending aorta) of this uptake was specified. Quantitative analyses were performed by measuring the maximum Standardized Uptake Value (SUV_max_) and target to background ratio (SUV_ratio_) on standardized European Association of Nuclear Medicine Research Ltd. (EARL) and non-EARL reconstructions using commercially available software (OsiriX MD version 7.5, Switzerland). SUV_max_ was measured in an automated volume of interest (VOI) around the PHV, which was visually verified to include the whole valve region. The SUV_ratio_ was then calculated as the ratio of the SUV_max_ and the mean SUV in the blood pool of the descending aorta, taking care not to include the vessel wall.

Myocardial suppression was scored as “fully suppressed” (no uptake), “low” (more than mediastinal uptake but less than in the liver), “intermediate” (more than liver uptake), “high focal” (much more than liver uptake, but focal), “high diffuse” (much more than liver uptake, diffuse).

#### Statistics

Descriptive statistics were used for analysis of the outcomes. For continuous variables, means and standard deviations (SD) were used in case of normal distribution. In case of non-normal distribution, medians and interquartile ranges (IQR) were used. The IQR and confidence interval (CI) were denoted in square brackets. Comparisons between groups were made using the Chi-square test for categorical variables. For continuous variables One-way Analysis of Variance (ANOVA) was used in case of normal distribution and Kruskal Wallis test in case of non-normal distribution. A significance level of *P* = 0.05 and 95% confidence intervals (CI) were used.

## Results

### Patients Characteristics and Classification

A total of 38 patients were initially included after signing written informed consent. One patient was excluded after failure to undergo the PET/CT scan due to scanner malfunction. Age (mean ± SD) of the 37 patients finally included in this study was 66 ± 8 years (group 1: 65 ± 7; group 2: 66 ± 8; group 3: 67 ± 10; *P* = 0.87) and most of the patients were male (n = 24, 65%) (group 1: n = 8; group 2: n = 10; group 3: n = 6; *P* = 0.15). There were 25 (68%) biological and 12 (32%) mechanical prosthetic valves, equally distributed between groups (*P* = 0.99). Surgical adhesives such as BioGlue that are known to be FDG-avid, were not used during any of the implantations. No patient was suspected of having endocarditis prior to the PET/CT scan. Patients were included in either group 1 (n = 12), group 2 (n = 12), or group 3 (n = 13). Due to logistic problems, 8 patients (group 1: n = 2; group 2 n = 3; group 3: n = 3) underwent the scan outside the time interval originally set-out for each group. The 2 patients in group 1 were scanned 2 and 5 days later than the maximum adjusted days (5 ± 1 week) for group 1. The 3 patients in group 2 were scanned 15, 22, and 38 days later and the 3 patients in group 3 were scanned 15, 23, and 36 days later than originally planned. Baseline characteristics for the overall population and the three groups are summarized in Table 2.

**Table 2:** Baseline characteristics of all patients and of patients in groups 1, 2, and 3

	All included patients	Group 1 (5 (± 1) weeks after PHV implantation)	Group 2 (12 (± 2) week after PHV implantation)	Group 3 (12 (± 2) months after PHV implantation)	*P* value***
Number of patients	37	12	12	13	
Age, mean±SD, years	66 ± 8	65 ± 7	66 ± 8	67 ± 10	0.87
Gender, n (%)					
Male	24(65)	8(67)	10(83)	6(46)	0.15
Female	13(35)	4(33)	2(17)	7(54)	
BMI, median [IQR], kg/m^2^	27 [24–29]	26 [23–30]	26 [25–28]	28 [25–30]	0.60
Days between PET/CT and PHV implantation, median [IQR], days	94 [42–360]	37 [35–42]	93 [87–109]	370 [356–430]	< 0.01
Laboratory results*					
Serum levels of leucocytes x10^9^ /L, mean ± SD	10.1 ± 2.3	9.8 ± 1.7	10.0 ± 2.3	10.5 ± 2.7	0.73
Serum levels of creatinine µmol/L, mean ± SD	71 ± 14	72 ± 16	76 ± 11	65 ± 13	0.13
Serum levels of glucose mmol/L, mean ± SD	5.4 ± 0.7	5.5 ± 0.6	5.5 ± 0.8	5.2 ± 0.8	0.46
Medical History, n (%)					
Hypertension	17 (46)	6 (50)	5 (42)	6 (46)	0.92
Atrial fibrillation	9 (24)	2 (17)	1 (8)	6 (46)	0.07
Hearth failure	1 (3)	0 (0)	1 (8)	0 (0)	0.34
Myocardial infarction	1 (3)	0 (0)	0 (0)	1 (8)	0.39
Prior thoracic surgery	3 (8)	1 (8)	1 (8)	1 (8)	0.999
PHV type, n (%)					0.99
Mechanical	12 (32)	4 (33)	4 (33)	4 (31)	
Biological	25 (68)	8 (67)	8 (67)	9 (69)	
Valve manufacturer, n (%)					0.62
St. Jude	9 (24)	3 (25)	2 (17)	4 (33)	
Carbomedics	3 (8)	1 (8)	2 (17)	0 (0)	
Perimount	25 (68)	8 (67)	8 (67)	9 (75)	
Valve Size (mm), n (%)					0.29
19	1 (3)	0 (0)	0 (0)	1 (8)	
21	5 (14)	3 (25)	0 (0)	2 (15)	
23	15 (41)	2 (17)	7 (58)	6 (46)	
25	12 (32)	6(50)	3 (25)	3 (23)	
27	4 (11)	1(8)	2 (17)	1 (8)	
Surgery, n (%)					
Concomitant CABG	14 (38)	4 (33)	6 (50)	4 (31)	0.57
Other concomitant procedure**	4 (11)	1 (8)	1 (8)	2 (15)	0.55
Use of surgical adhesives	0 (0)	0 (0)	0 (0)	0 (0)	1.0

*BMI*, body mass index; *CABG*, coronary artery bypass grafting; *PHV*, prosthetic heart valve; *PET=CT*, positron emission tomography with computed tomography

*Serum Leucocytes and Creatinine levels were measured as part of clinical practice ± 5days after valve implantation and serum glucose levels were measured on the day of 18F-FDG PET/CT scan

**Four patients underwent a concomitant procedure with the aortic PHV implantation containing two patients with a MAZE procedure, one patient with a myectomy and additional mitral valve replacement and one patient with pulmonary vene ablation on both sides

***Statistical difference between the three groups 1, 2, and 3

### ^18^F-FDG PET/CT Findings

The median time between PHV implantation and ^18^F-FDG PET/CT was 37 [IQR 35–42], 93 [IQR 87 to 109], and 370 [IQR 356 to 430] days for group 1, 2, and 3 respectively (*P* < 0.01). Median ^18^F-FDG dosage was 166 [IQR 145 to 183] MBq and not significantly different between the groups (*P* = 0.16). Preparation according to carbohydrate diet protocol was followed by 36/37 (97%) patients. Three patients had fasted less than 12 hours prior to the scan, 1 patient failed to follow the low carbohydrate diet and 1 patient inadvertently received a double amount of ^18^F-FDG activity. Myocardial ^18^F-FDG uptake was scored as “fully suppressed” in 18/37 (49%) and as intermediate or less in 29/37 (78%) patients. One patient was scored as focal high and 7 patients as diffuse high myocardial uptake. The interpretation of one scan was hampered due to the diffuse high myocardial FDG uptake.

**Figure 1:**
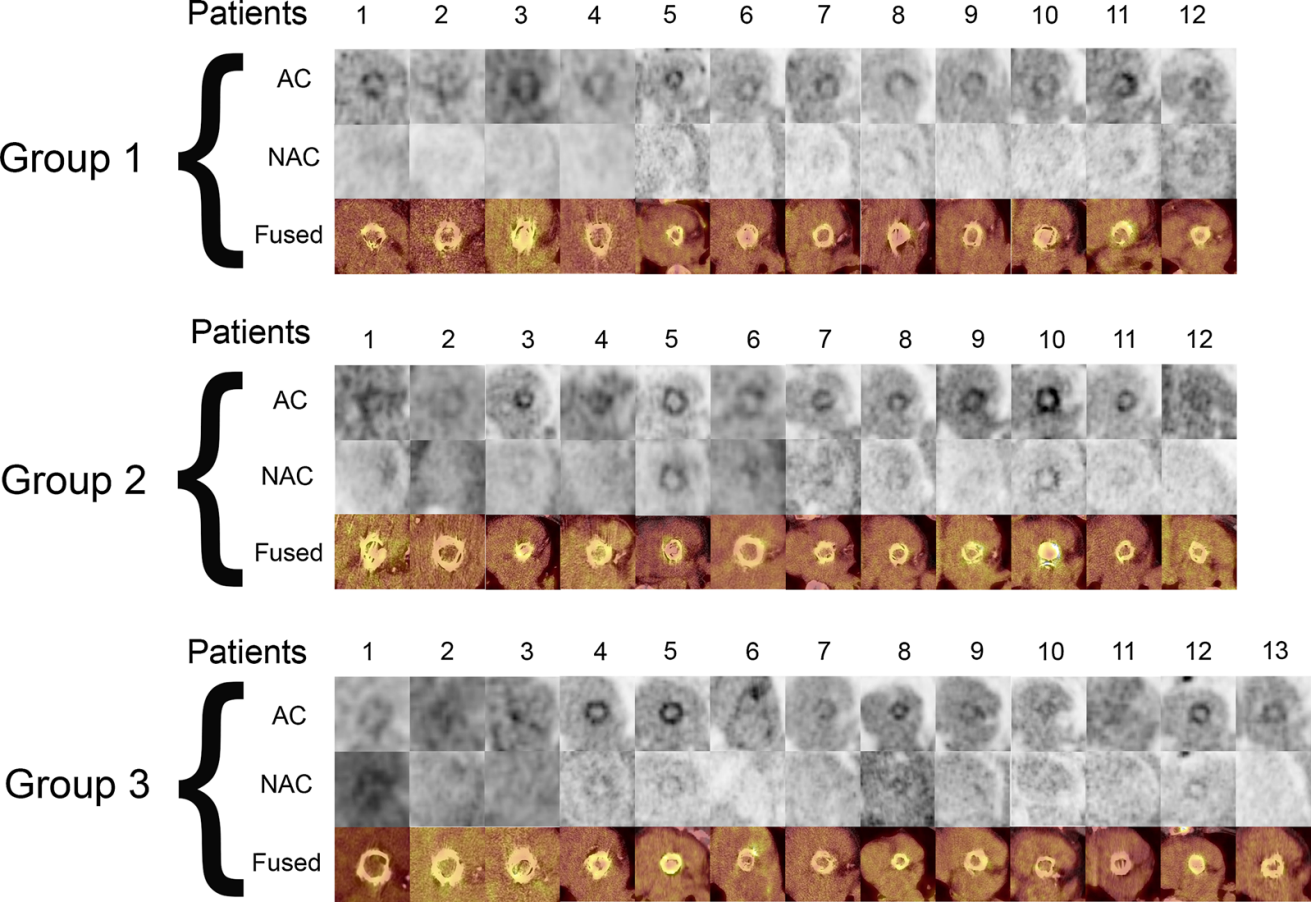
^18^F-FDG uptake around the PHV on reconstructed views in plane with the PHV of attenuation-corrected (AC) images, non-attenuation-corrected (NAC) and fused attenuation-corrected images with CT in all patients. Scaling was set the same for all AC images (0-7MBq)

The QVSH around the PHV was scored as follows for group 1: low in 8/12 (67%) and intermediate in 4/12 (33%) patients; group 2: 7/12 (58%) low and 5/12 (42%) intermediate and for group 3: 8/13 (62%) low and 5/13 (38%) intermediate. Comparison between groups showed no significant difference in QVSH (*P* = 0.91). The distribution of ^18^F-FDG uptake was circular in most cases (78%) and not significantly different between the 3 groups (*P* = 0.50). The ^18^F-FDG uptake around the PHVs on a reconstructed view in the PHV plane of attenuation-corrected images, non-attenuation-corrected and fused attenuation-corrected images with CT of all patients is shown in Figure 1.

**Figure 2:**
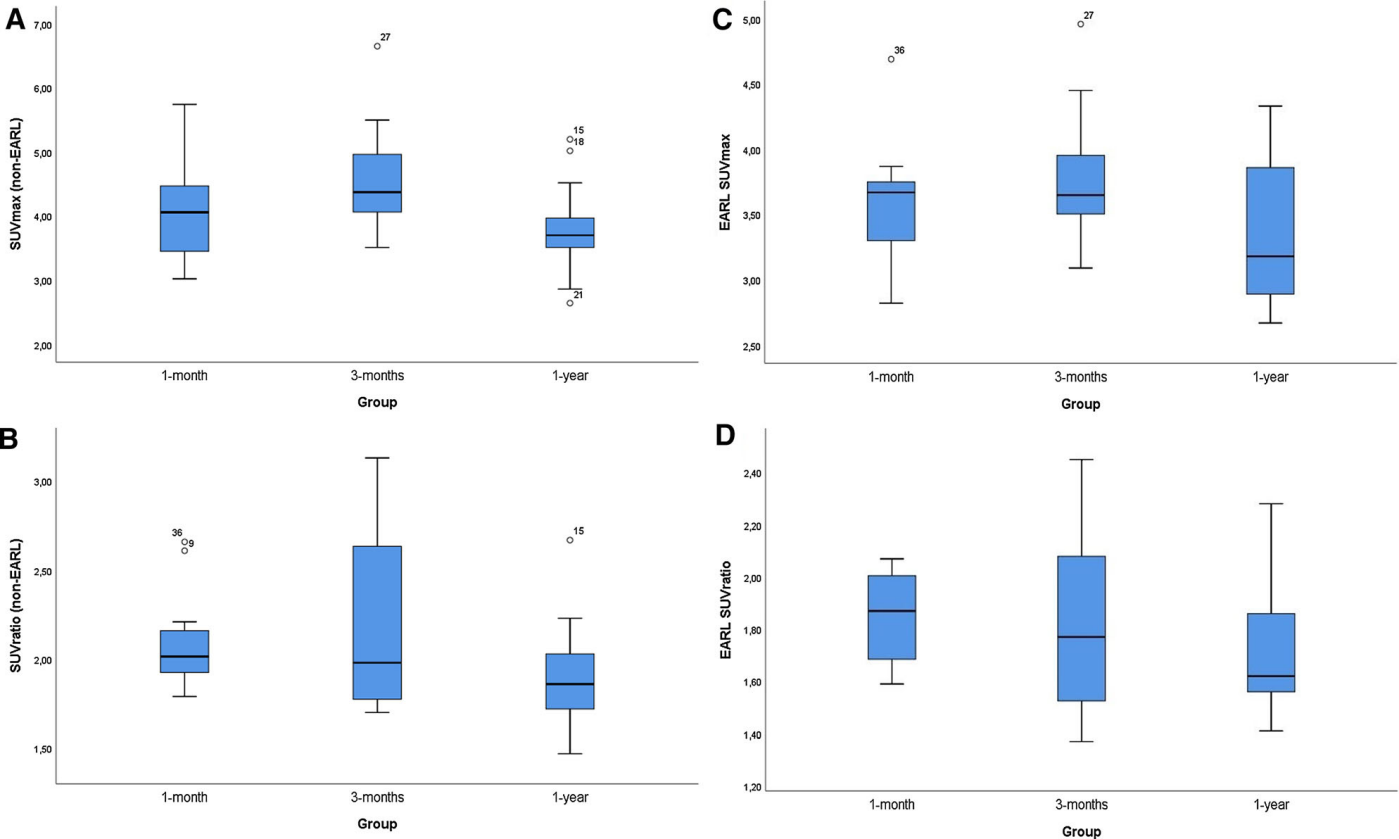
Boxplots of the non-EARL (**A**, **B**) and EARL (C,D) SUV_max_ and SUV_ratio_ measurement distribution in each group. The dots indicated as “15”, “18”, “21”, “27” (**A**) “9”, “15”, “36” (**B**) “27” and “36” (**C**) are outliers in the SUV_max_ and SUV_ratio_ measurements

Quantitative analyses on the non-EARL attenuation-corrected images showed a SUV_max_ of 4.1 ± 0.8 (mean ± SD) and a median[IQR] SUV_ratio_ of 2.0 [1.8 to 2.2] for all included patients. The SUV_max_ around the PHV was 4.1 ± 0.7, 4.6 ± 0.9, and 3.8 ± 0.7 (mean ± SD) in group 1, 2, and 3 respectively, with no significant difference between the 3 groups (p = 0.08). The median[IQR] SUV_ratio_ around the PHV was 2.0 [1.9 to 2.2], 2.0 [1.8 to 2.6], and 1.9 [1.7 to 2.0] with no significant difference between the three groups (*P* = 0.81) (Table 3). Quantitative analyses on the EARL reconstruction images showed an average SUV_max_ and SUV_ratio_ of 3.6 ± 0.5 and 1.8 ± 0.3 (mean ± SD), respectively. SUV_max_ around the PHV was 3.6 ± 0.5, 3.8 ± 0.5 and 3.3 ± 0.6 (mean ± SD) in group 1, 2 and 3 respectively, with no significant difference between the 3 groups (*P* = 0.14). Likewise, the SUV_ratio_ around the PHV was 1.8 ± 0.2, 1.8 ± 0.3, and 1.7 ± 0.3(mean ± SD) with no significant difference between the three groups either (*P* = 0.41). The minimum and maximum measured SUV_ratio_ in the study population was 1.4 and 2.5, respectively. EARL SUV_ratio_ was < 2.3 in 97% and < 2.1 in 92% of the cases. The distribution of non-EARL and EARL SUV_max_ and SUV_ratio_ are demonstrated in Figure 2.

**Table 3:** ^18^F-FDG PET/CT findings for all patients and for each patient per group

	All included patients	Group 1 (5 (± 1) weeks after PHV implantation)	Group 2 (12 (± 2) week after PHV implantation)	Group 3 (12 (± 2) months after PHV implantation)	*P* value*
Number of patients	37	12	12	13	
FDG dose, MBq/kg, m[IQR]	166 [145–183]	160 [134–175]	172 [156–181]	180 [140–188]	0.16
Time between FDG dose and start scan (min), m[IQR]	60 [58–64]	59 [57–63]	60 [59–63]	60 [58–66]	0.82
Serum levels of glucose mmol/L (mean ± SD)	5.4 ± 0.7	5.5 ± 0.6	5.5 ± 0.8	5.2 ± 0.8	0.47
Preparation according to carbohydrate diet protocol, n(%)	36 (97)	11 (92)	12 (100)	13 (100)	0.34
Myocardial suppression, n (%)					0.70
Fully suppressed	18 (49)	7 (58)	5 (42)	6 (46)	
Low uptake	1 (3)	1 (8)	0 (0)	0 (0)	
Intermediate uptake	10 (27)	2 (17)	3 (25)	5 (38)	
High focal uptake	1 (3)	0 (0)	1 (8)	0 (0)	
High diffuse uptake	7 (19)	2 (17)	3 (25)	2 (15)	
Elevated uptake elsewhere in the body, n (%)	21 (57)	7 (58)	9 (75)	5 (38)	0.34
Visual score PHV (QVSH), n (%)					0.91
None	0 (0)	0 (0)	0 (0)	0 (0)	
Low	23 (62)	8 (67)	7 (58)	8 (62)	
Intermediate	14 (38)	4 (33)	5 (42)	5 (38)	
High	0 (0)	0 (0)	0 (0)	0 (0)	
Specific location FDG uptake, n (%)					0.50
Former LCC	1 (3)	0 (0)	1 (8)	0 (0)	
Former NCC	1 (3)	1 (8)	0 (0)	0 (0)	
Circular	29 (78)	8 (67)	9 (75)	12 (92)	
Struts only	5 (14)	2 (17)	2 (17)	1 (8)	
Multiple	1 (3)	1 (8)	0 (0)	0 (0)	
SUV_max_ PHV (mean ± SD)	4.1 ± 0.8	4.1 ± 0.7	4.6 ± 0.9	3.8 ± 0.7	0.08
SUV_ratio_ PHV m[IQR]	2.0 [1.8–2.2]	2.0 [1.9–2.2]	2.0 [1.8–2.6]	1.9 [1.7–2.0]	0.81
EARL SUV_max_ PHV (mean ± SD)	3.6 ± 0.5	3.6 ± 0.5	3.8 ± 0.5	3.3 ± 0.6	0.14
EARL SUV_ratio_ PHV (mean ± SD)	1.8 ± 0.3	1.8 ± 0.2	1.8 ± 0.3	1.7 ± 0.3	0.41

*PHV*, prosthetic heart valve; *MBq/kg*, megabecquerel/kilograms; *QVSH*, qualification visual score of hypermetabolism; *LCC*, left coronary cusp; *NCC*, non coronary cusp; *SUVmax*, maximum standardized uptake value; *SUVratio*, standardized uptake value ratio (Target to background ratio); *EARL*, European Association of nuclear medicine Research Ltd

*Statistical difference between the three groups 1, 2 and 3

Elevated ^18^F-FDG uptake elsewhere in the body was seen in 21/37 (57%) of patients and was not significantly different between the 3 groups (*P* = 0.18). This elevated ^18^F-FDG uptake was mainly seen in the thoracic lymphnodes 9/21 (38%) and considered physiological. Other areas of elevated uptake consisted of costal fractures 3/21 (14%), pleural uptake (possible pulmonary nodule) 2/21 (10%), acromioclavicular joint (due to degeneration) 2/21 (10%), thyroid (possible hyperthyroidism) 1/21 (5%), arytenoid (physiological) 1/21 (5%), possible pathological oesophageal uptake 2/21 (10%), diffuse in muscles 1/21 (5%), and focal uptake due to a surgical clip.

## Discussion

The present study shows that patients with non-infected aortic PHV have similar low to intermediate mostly circular ^18^F-FDG uptake around the PHV at 5, 12 and 52 weeks after implantation and a mean ± SD SUV_max_ of 4.1 ± 0.8 and a median[IQR] SUV_ratio_ of 2.0[1.8 to 2.2].

Nowadays, ^18^F-FDG PET/CT is an important diagnostic method in suspected PHV endocarditis, especially in cases where the diagnosis cannot be confirmed with transthoracic (TTE) or transesophageal echocardiography (TEE). However, in patients with a recent PHV implantation (< 3 months), the use of ^18^F-FDG PET/CT is not advised due to possible false positive findings caused by post-surgical inflammation.^2^ Misinterpretation of ^18^F-FDG PET/CT findings could have major inappropriate therapeutic consequences. Patients may be treated while this is not necessary and counterwise not be treated while this is obligatory. Therefore, caution with the interpretation of ^18^F-FDG PET/CT in the early weeks after PHV implantation is advised, especially in cases of complicated surgery. In such cases, the inflammation response due to the complications could be severe and cause non-diagnostic or false positive ^18^F-FDG PET/CT results. It is therefore crucial to be able to recognize normal ^18^F-FDG distribution patterns and establish a quantitative cut-off value for pathological ^18^F-FDG uptake around the PHV.

Quantitative measurements of ^18^F-FDG uptake around the PHV in our study demonstrated a median[IQR] SUV_ratio_ of 2.0 [1.9 to 2.2] for patients at 5 weeks after surgery, with no statistically significant difference compared to 3 months and 1 year (2.0 [1.8 to 2.6] and 1.9 [1.7 to 2.0], respectively; *P* = 0.81). These results corroborate the scarce known literature about this matter. Mathieu et al.^7^ reported on a retrospectively included group of 35 patients with aortic PHVs who underwent a PET/CT scan < 3 months and > 3 months after PHV implantion for either oncological imaging, large vessel vasculitis or suspicion of prosthetic valve endocarditis that was subsequently rejected, and found a median SUV_max_ of 3.6 [2.1 to 8.0, range] and a median SUV_ratio_ of 1.9 [1.3 to 6.6, range] on non-EARL attenuation-corrected images. No significant difference in SUV_max_ and SUV_ratio_ between the PHVs implanted < 3 months and those that were implanted > 3 months prior to the PET/CT scan was found.^7^ However, these results should be interpreted with some caution because: (1) the patient population was diverse and included patients with vasculitis and a rejected suspicion of endocarditis and (2) 24/35 (69%) of the valves were implanted more than 1 year ago. The authors also reported a much higher median SUVmax of 4.7 and SUV_ratio_ of 2.7 in the patients with vasculitis compared to the other groups.^7^ Roque et al.^8^ have recently presented a prospective analysis of ^18^F-FDG uptake at 3 different time points in the first year after PHV implantation. The study method had similarities with our study, but there were some differences. Roque et al. included also patients post mitral valve implantation, and each patient received 3 times a PET/CT scan in the time periods of 1, 6, and 12 months after valve implantation. Despite these differences, their results also showed no significant difference in ^18^F-FDG uptake between scans made in the three different time periods and their conclusion that the three months safety period should be reconsidered is in line with our conclusion.

Recently, in a retrospectively collected cohort of 243 patients, we found that the optimal diagnostic cut-off value to diagnose PHV endocarditis for the EARL-standardized SUV_ratio_ was > 2.0.^3^ In our current study the maximum measured EARL SUV_ratio_ was 2.5 and 97% of scans had an EARL SUV_ratio_ of less than 2.3, indicating that the cut-off value might be slightly higher than the > 2.0 reported earlier by Swart et al. in the first year after PHV implantation^3^ and also higher than the mean values reported by Mathieu et al.^7^

**Figure 3:**
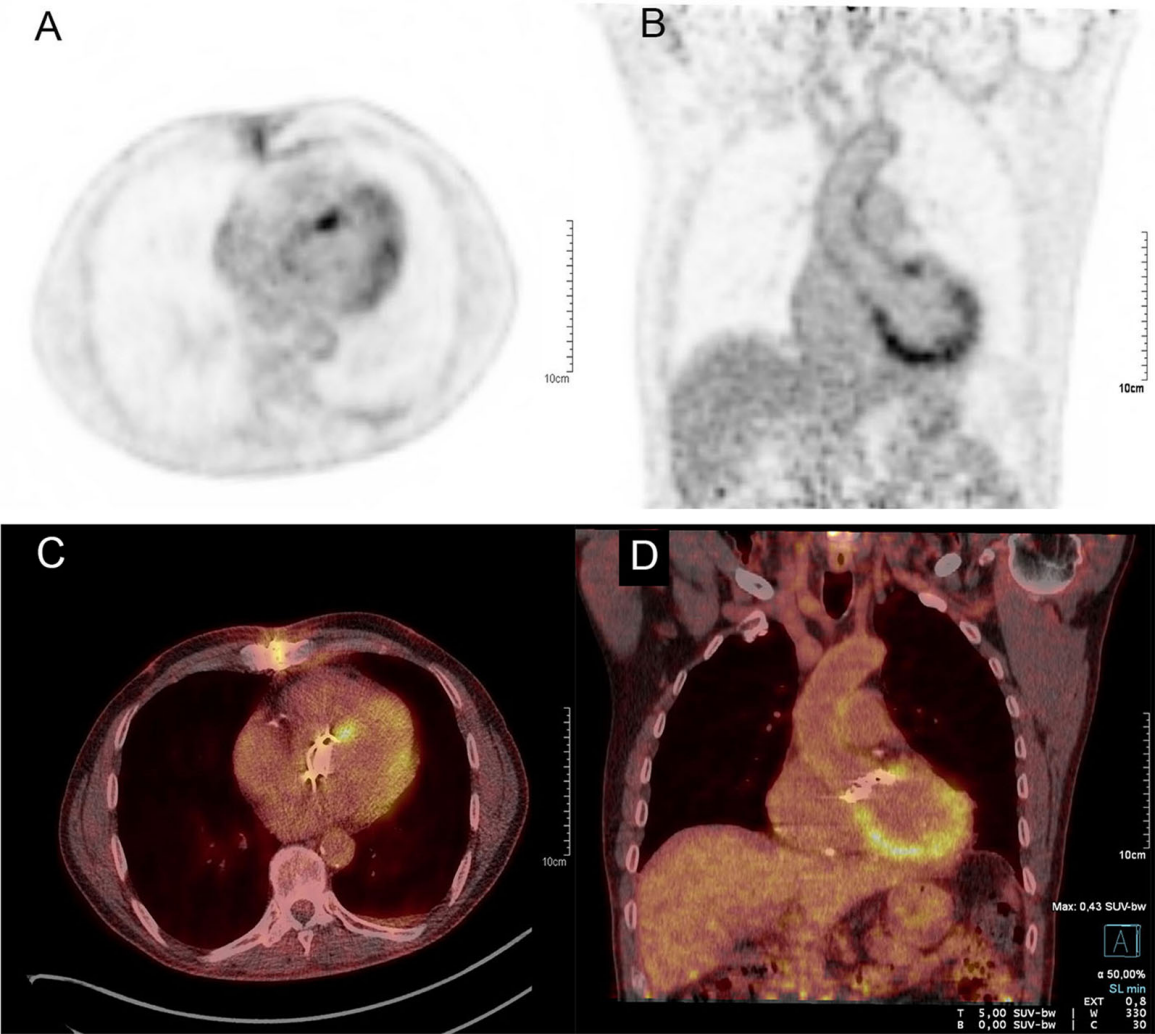
Attenuation-corrected ^18^F-FDG PET images (**A**, **B**) and fused images (**C**, **D**) of a patient with a high level of myocardial ^18^F-FDG uptake making correct measurements of the SUV values more difficult

In our current study, we found only diffuse ^18^F-FDG uptake around the PHV with mostly a circular pattern (29/37, 78%) and without focal enhancement. The distribution of ^18^F-FDG can differ widely and its definition is still unclear; however, some of the uptake patterns (eg. diffuse around PHV without focal enhancement) have been associated with physiological uptake after PHV implantation.^7^ Furthermore, physiological myocardial uptake during ^18^F-FDG PET/CT can mask adjacent abnormal ^18^F-FDG uptake around the PHV. Therefore a preparatory low carbohydrate diet that may be supplemented by an intravenous injection of heparin is necessary for reducing myocardial ^18^F-FDG uptake in order to avoid false positive ^18^F-FDG PET/CT results.^9^–^12^ In our study, one patient had failed to follow the prepatory low carbohydrate diet and demonstrated indeed a high level of myocardial ^18^F-FDG uptake making correct measurement of the SUV values more difficult (Figure 3).

Our study has some limitations. Eight patients (group 1: n = 2, group 2: n = 3, and group 3: n = 3) received the scan somewhat later than the time frame adjusted for each group. This was due to logistic reasons. Another limitation of this study was that the scan was performed once in every patient and not multiple times in the same patient to actually see a change over time in the uptake patterns and SUV values. This approach was not deemed feasible due to the high radiation dose of multiple PET/CT scans in individual healthy patients this would imply. Furthermore, our study population only included patients with an aortic prosthetic valve, and hence we cannot draw any conclusion regarding normal ^18^F-FDG findings for prosthetic valve in other locations or regarding combined aortic valve and ascending aorta replacements (e.g., Bentall procedure). Excluding obese patients and patients with diabetes mellitus could also be seen as a limitation to the applicability of our results. Both conditions can affect the healing process following surgery and could therefore potentially impact ^18^F-FDG uptake. However, in order to prevent inadequate glucose levels prior to the PET and restrict the radiation exposure to patients, the exclusion of these patients was necessary. In total 51% of the patients did not have fully suppressed myocardium and this could be seen as a potential confounder to the qualitative and quantitative ^18^F-FDG measurements.

Although the measurements done by the nuclear medicine physicians were carefully done not to include myocardial uptake, this could not always have been prevented. Thus, this could be seen as a limitation of our study.

In conclusion, non-infected aortic PHV have similar low to intermediate mostly circular perivalvular ^18^F-FDG uptake at 5, 12, and 52 weeks after implantation and an average SUV_max_ of 4.1 ± 0.8 and a median[IQR] SUV_ratio_ of 2.0 [1.8 to 2.2]. These normal ^18^F-FDG uptake values and patterns provide further evidence that ^18^F-FDG PET-CT can be used as a diagnostic tool for the detection of endocarditis even shortly after aortic PHV implantation and the recommendation to not perform PET-CT within the first three months after PHV implantation in the 2015 ESC guidelines for the management of infective endocarditis should be reconsidered.

## New Knowledge Gained

Our study supports previous observations on the normal perivalvular ^18^F-FDG uptake within the first year after PHV implantation and showed no significant difference in ^18^F-FDG uptake at 5 weeks, 12 weeks, or 52 weeks after implantation. These findings may help clinicians to differentiate between normal and pathological perivalvular ^18^F-FDG uptake and suggest the use of ^18^F-FDG PET/CT as an extra imaging tool in the diagnostic workup of patients with recent aortic PHV implantation that are suspected of PHV endocarditis.

## Electronic supplementary material

Below is the link to the electronic supplementary material.

Electronic supplementary material 1 (PPTX 156 kb)

Electronic supplementary material 2 (M4A 8025 kb)

## Abbreviations

PHV: Prosthetic heart valve
^18^F-FDG: ^18^F-Fluorodeoxyglucose
PET: Positron emission tomography
ESC: European Society of Cardiology
QVSH: Qualification visual score for hypermetabolism
EARL: European Association of Nuclear Medicine Research Ltd
TTE: Transthoracic echocardiography
TEE: Transesophageal echocardiography

## References

[1] Habib G, Derumeaux G, Avierinos JF, Casalta JP, Jamal F, Volot F (1999). Value and limitations of the Duke criteria for the diagnosis of infective endocarditis. J Am Coll Cardiol.

[2] Habib G, Lancellotti P, Antunes MJ, Bongiorni MG, Casalta JP, Del Zotti F (2015). 2015 ESC Guidelines for the management of infective endocarditis: The Task Force for the Management of Infective Endocarditis of the European Society of Cardiology (ESC). Endorsed by: European Association for Cardio-Thoracic Surgery (EACTS), the European Association of Nuclear Medicine (EANM). Eur Heart J.

[3] Swart LE, Gomes A, Scholtens AM, Sinha B, Tanis W, Lam M (2018). Improving the diagnostic performance of (18)F-Fluorodeoxyglucose positron-emission tomography/computed tomography in prosthetic heart valve endocarditis. Circulation.

[4] Juneau D, Golfam M, Hazra S, Erthal F, Zuckier LS, Bernick J (2018). Molecular Imaging for the diagnosis of infective endocarditis: A systematic literature review and meta-analysis. Int J Cardiol.

[5] Mahmood M, Kendi AT, Ajmal S, Farid S, O’Horo JC, Chareonthaitawee P (2019). Meta-analysis of 18F-FDG PET/CT in the diagnosis of infective endocarditis. J Nucl Cardiol.

[6] Scholtens AM, Budde RPJ, Lam M, Verberne HJ (2017). FDG PET/CT in prosthetic heart valve endocarditis: There is no need to wait. J Nucl Cardiol.

[7] Mathieu C, Mikail N, Benali K, Iung B, Duval X, Nataf P (2017). Characterization of (18)F-Fluorodeoxyglucose uptake pattern in noninfected prosthetic heart valves. Circ Cardiovasc Imaging.

[8] Roque A, Pizzi MN, Fernandez-Hidalgo N, Permanyer E, Cuellar-Calabria H, Romero-Farina G (2019). Morpho-metabolic post-surgical patterns of non-infected prosthetic heart valves by [18F]FDG PET/CTA: “normality” is a possible diagnosis. Eur Heart J Cardiovasc Imaging.

[9] Coulden R, Chung P, Sonnex E, Ibrahim Q, Maguire C, Abele J (2012). Suppression of myocardial 18F-FDG uptake with a preparatory “Atkins-style” low-carbohydrate diet. Eur Radiol.

[10] Kapoor V, McCook BM, Torok FS (2004). An introduction to PET-CT imaging. Radiographics.

[11] Scholtens AM, Swart LE, Verberne HJ, Tanis W, Lam MG, Budde RP (2016). Confounders in FDG-PET/CT imaging of suspected prosthetic valve endocarditis. JACC Cardiovasc Imaging.

[12] Scholtens AM, Verberne HJ, Budde RP, Lam MG (2016). Additional heparin preadministration improves cardiac glucose metabolism suppression over low-carbohydrate diet alone in (1)(8)F-FDG PET imaging. J Nucl Med.

