# Supplementary material for: Normal imaging findings after aortic valve implantation on 18F-Fluorodeoxyglucose positron emission tomography with computed tomography
Source: J Nucl Cardiol. 2020 Jan 23;28(5):2258–68. doi: 10.1007/s12350-019-02025-y (PMC8648629; doi:10.1007/s12350-019-02025-y)
Supplement: Supplementary file 1 — Electronic supplementary material 1 (PPTX 156 kb) [file 12350_2019_2025_MOESM1_ESM.pptx]

## Slide 1
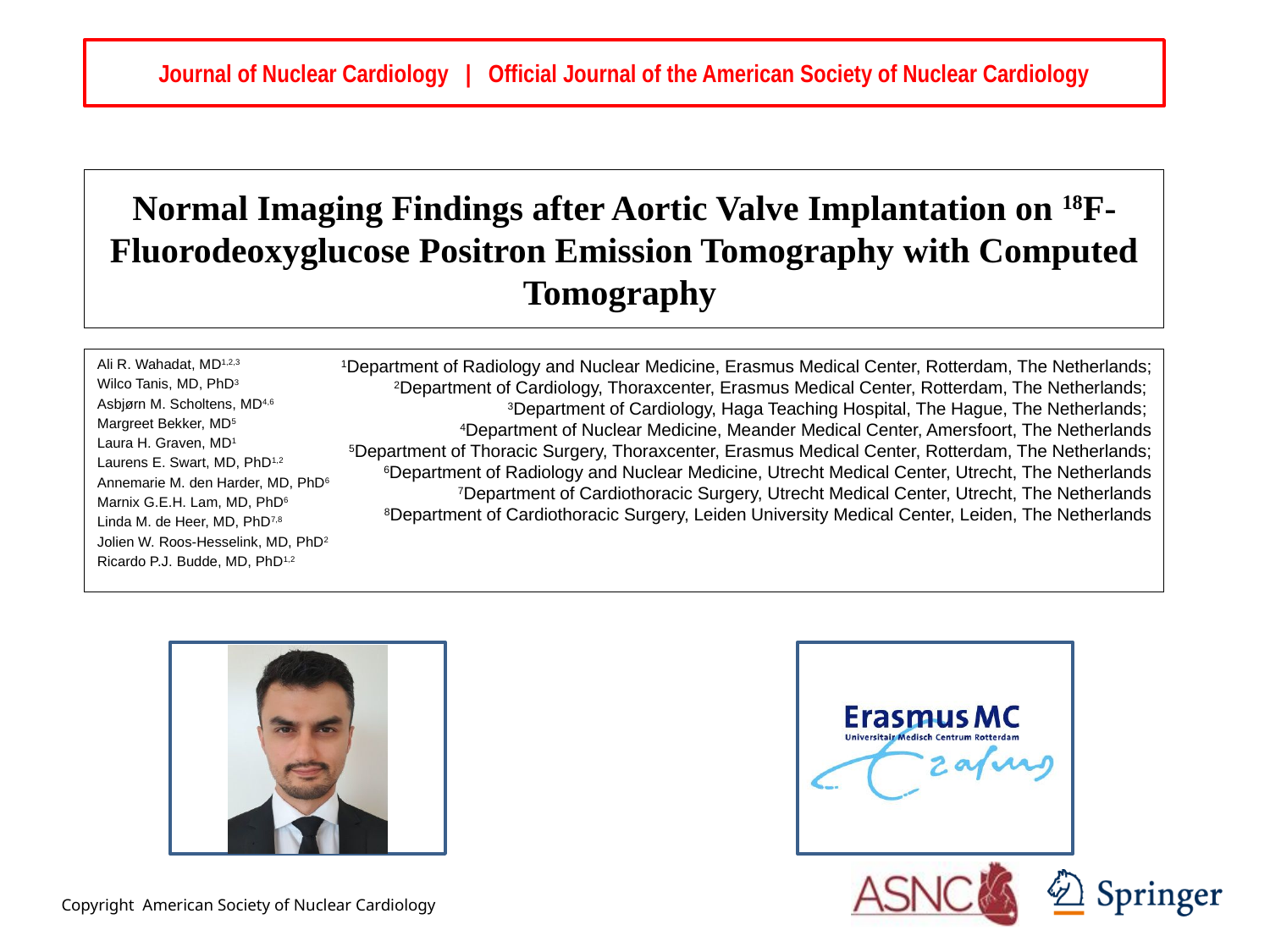

Journal of Nuclear Cardiology | Official Journal of the American Society of Nuclear Cardiology
# Normal Imaging Findings after Aortic Valve Implantation on 18F-Fluorodeoxyglucose Positron Emission Tomography with Computed Tomography
Ali R. Wahadat, MD1,2,3
Wilco Tanis, MD, PhD3
Asbjørn M. Scholtens, MD4,6
Margreet Bekker, MD5
Laura H. Graven, MD1
Laurens E. Swart, MD, PhD1,2
Annemarie M. den Harder, MD, PhD6
Marnix G.E.H. Lam, MD, PhD6
Linda M. de Heer, MD, PhD7,8
Jolien W. Roos-Hesselink, MD, PhD2
Ricardo P.J. Budde, MD, PhD1,2
1Department of Radiology and Nuclear Medicine, Erasmus Medical Center, Rotterdam, The Netherlands;
2Department of Cardiology, Thoraxcenter, Erasmus Medical Center, Rotterdam, The Netherlands;
3Department of Cardiology, Haga Teaching Hospital, The Hague, The Netherlands;
4Department of Nuclear Medicine, Meander Medical Center, Amersfoort, The Netherlands
5Department of Thoracic Surgery, Thoraxcenter, Erasmus Medical Center, Rotterdam, The Netherlands;
6Department of Radiology and Nuclear Medicine, Utrecht Medical Center, Utrecht, The Netherlands
7Department of Cardiothoracic Surgery, Utrecht Medical Center, Utrecht, The Netherlands
8Department of Cardiothoracic Surgery, Leiden University Medical Center, Leiden, The Netherlands
Copyright American Society of Nuclear Cardiology

## Slide 2
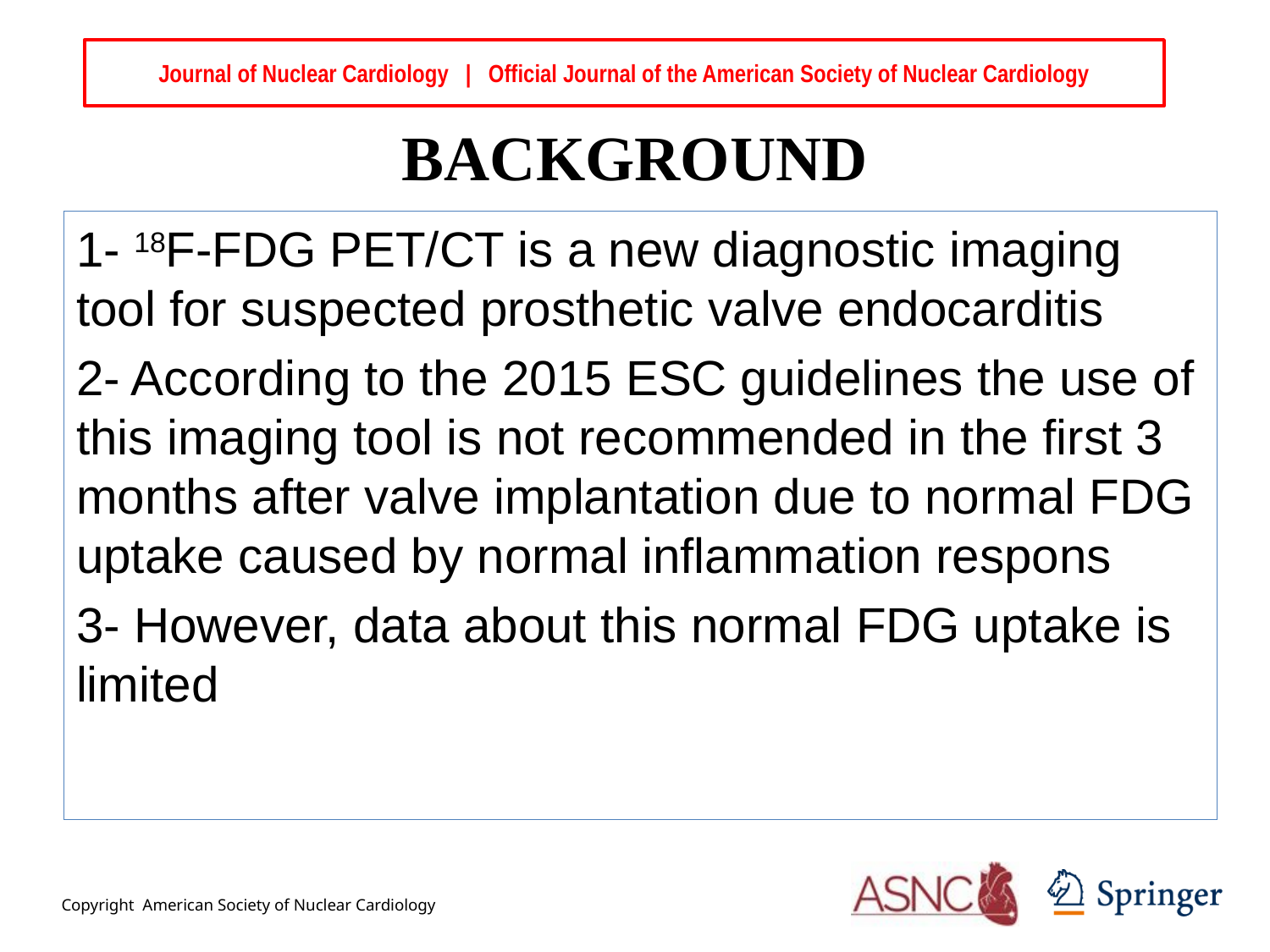

Journal of Nuclear Cardiology | Official Journal of the American Society of Nuclear Cardiology
# BACKGROUND
1- 18F-FDG PET/CT is a new diagnostic imaging tool for suspected prosthetic valve endocarditis
2- According to the 2015 ESC guidelines the use of this imaging tool is not recommended in the first 3 months after valve implantation due to normal FDG uptake caused by normal inflammation respons
3- However, data about this normal FDG uptake is limited
Copyright American Society of Nuclear Cardiology

## Slide 3
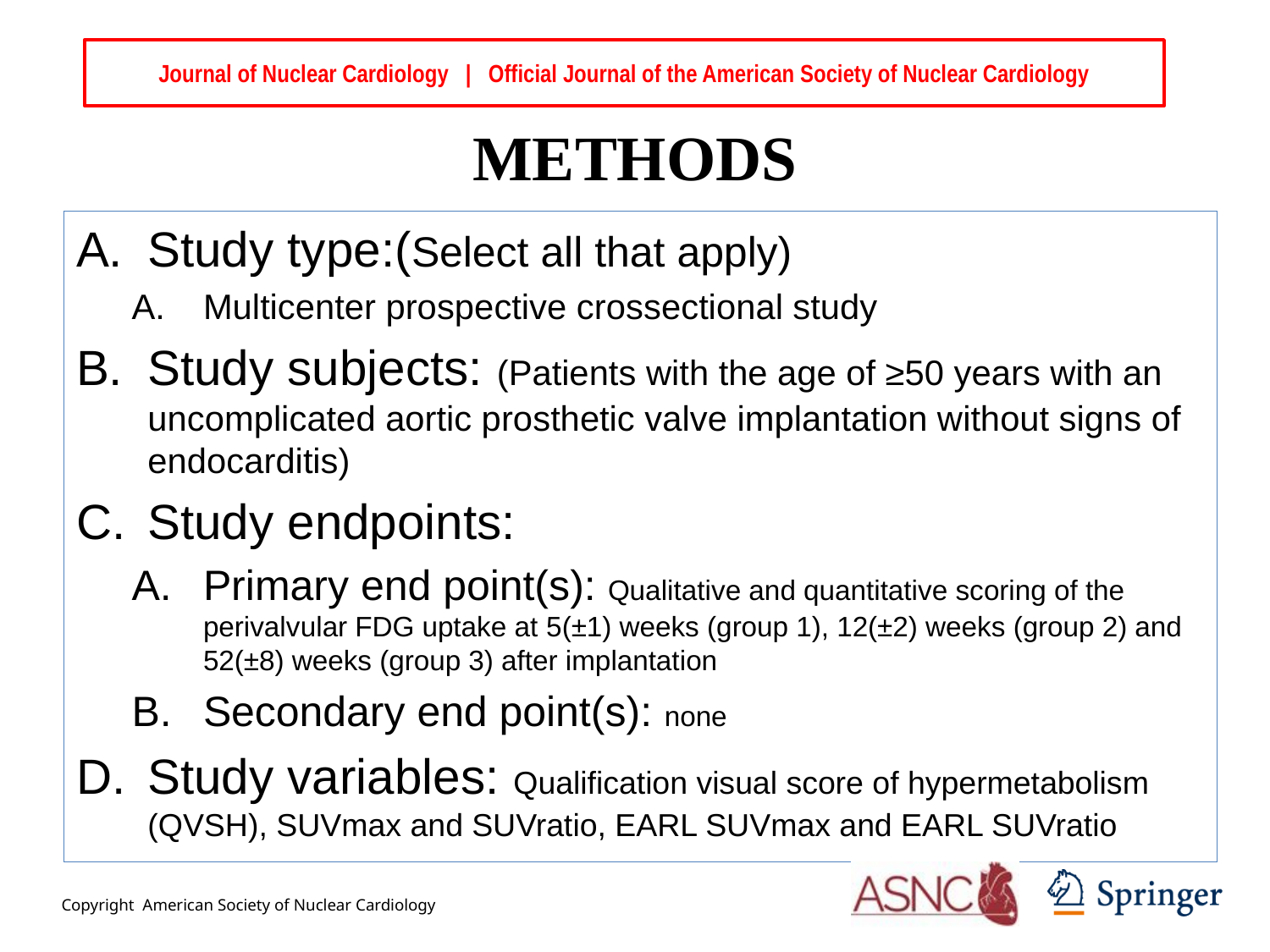

Journal of Nuclear Cardiology | Official Journal of the American Society of Nuclear Cardiology
# METHODS
Study type:(Select all that apply)
Multicenter prospective crossectional study
Study subjects: (Patients with the age of ≥50 years with an uncomplicated aortic prosthetic valve implantation without signs of endocarditis)
Study endpoints:
Primary end point(s): Qualitative and quantitative scoring of the perivalvular FDG uptake at 5(±1) weeks (group 1), 12(±2) weeks (group 2) and 52(±8) weeks (group 3) after implantation
Secondary end point(s): none
Study variables: Qualification visual score of hypermetabolism (QVSH), SUVmax and SUVratio, EARL SUVmax and EARL SUVratio
Copyright American Society of Nuclear Cardiology

## Slide 4
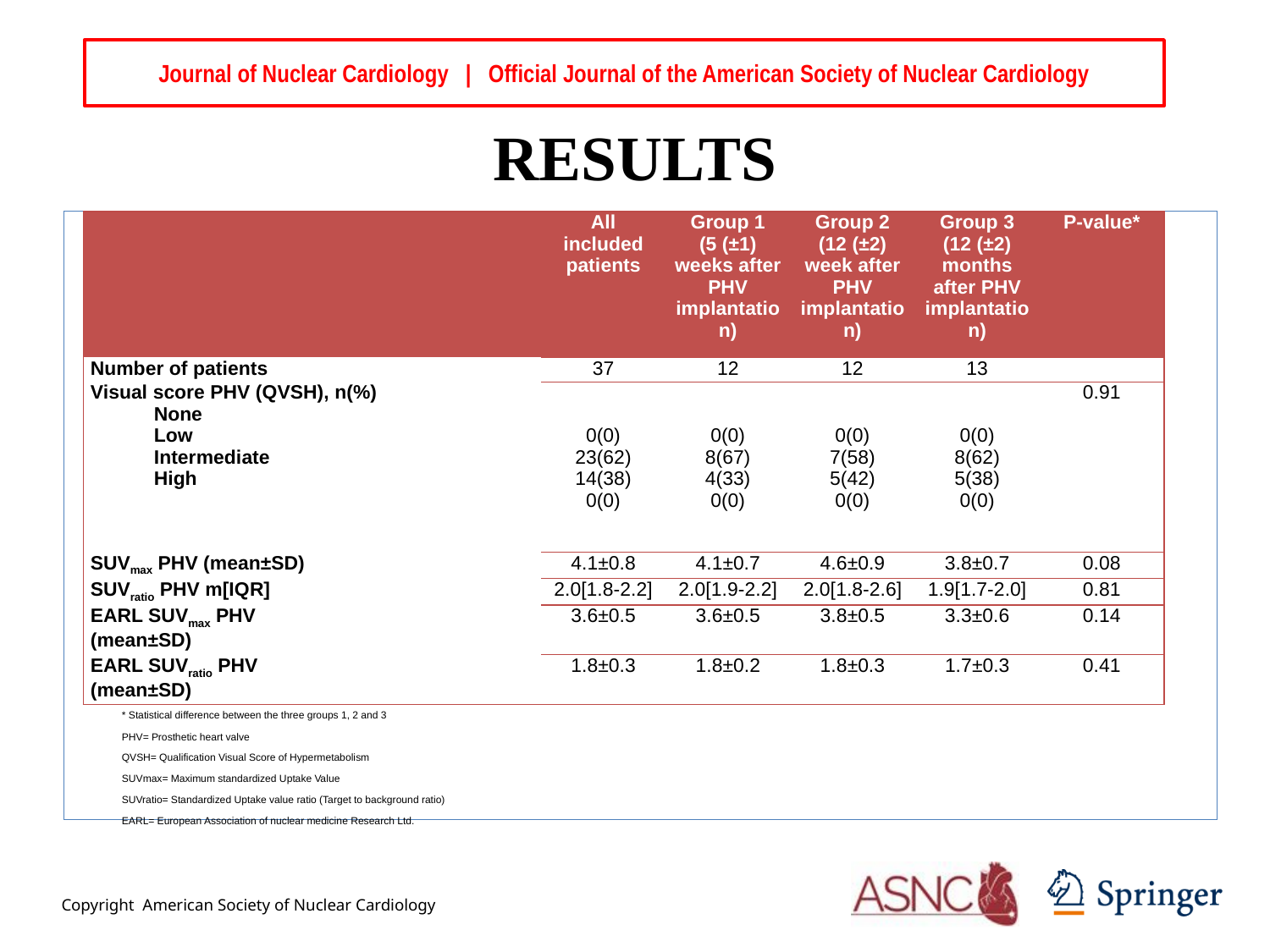

Journal of Nuclear Cardiology | Official Journal of the American Society of Nuclear Cardiology
# RESULTS
| | All included patients | Group 1 (5 (±1) weeks after PHV implantation) | Group 2 (12 (±2) week after PHV implantation) | Group 3 (12 (±2) months after PHV implantation) | P-value\* |
| --- | --- | --- | --- | --- | --- |
| Number of patients | 37 | 12 | 12 | 13 | |
| Visual score PHV (QVSH), n(%) None Low Intermediate High | 0(0) 23(62) 14(38) 0(0) | 0(0) 8(67) 4(33) 0(0) | 0(0) 7(58) 5(42) 0(0) | 0(0) 8(62) 5(38) 0(0) | 0.91 |
| SUVmax PHV (mean±SD) | 4.1±0.8 | 4.1±0.7 | 4.6±0.9 | 3.8±0.7 | 0.08 |
| SUVratio PHV m[IQR] | 2.0[1.8-2.2] | 2.0[1.9-2.2] | 2.0[1.8-2.6] | 1.9[1.7-2.0] | 0.81 |
| EARL SUVmax PHV (mean±SD) | 3.6±0.5 | 3.6±0.5 | 3.8±0.5 | 3.3±0.6 | 0.14 |
| EARL SUVratio PHV (mean±SD) | 1.8±0.3 | 1.8±0.2 | 1.8±0.3 | 1.7±0.3 | 0.41 |
* Statistical difference between the three groups 1, 2 and 3
PHV= Prosthetic heart valve
QVSH= Qualification Visual Score of Hypermetabolism
SUVmax= Maximum standardized Uptake Value
SUVratio= Standardized Uptake value ratio (Target to background ratio)
EARL= European Association of nuclear medicine Research Ltd.
Copyright American Society of Nuclear Cardiology

## Slide 5
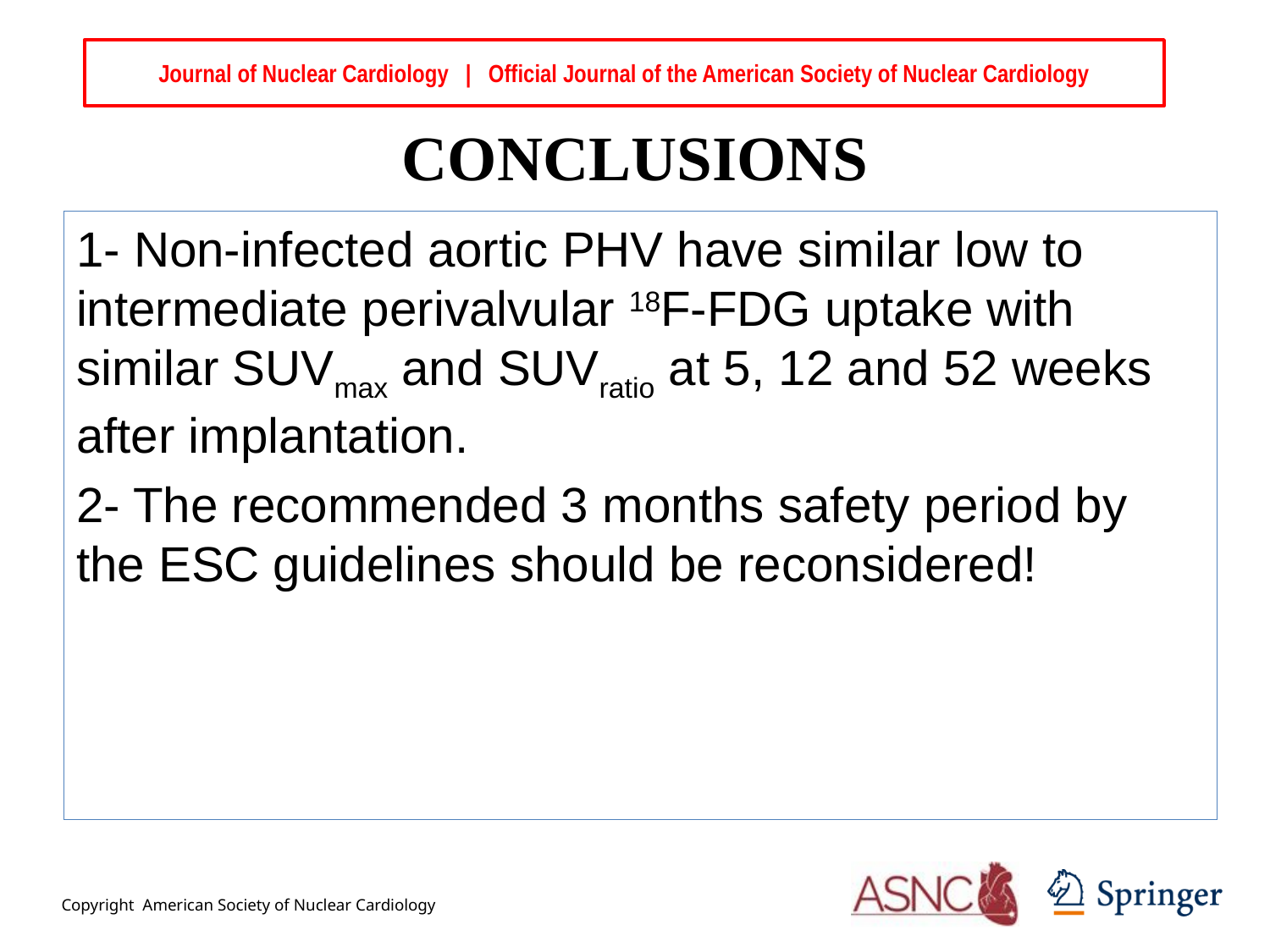

Journal of Nuclear Cardiology | Official Journal of the American Society of Nuclear Cardiology
# CONCLUSIONS
1- Non-infected aortic PHV have similar low to intermediate perivalvular 18F-FDG uptake with similar SUVmax and SUVratio at 5, 12 and 52 weeks after implantation.
2- The recommended 3 months safety period by the ESC guidelines should be reconsidered!
Copyright American Society of Nuclear Cardiology
